# Genomic predictions based on animal models using genotype imputation on a national scale in Norwegian Red cattle

**DOI:** 10.1186/s12711-015-0159-8

**Published:** 2015-10-13

**Authors:** Theo H. E. Meuwissen, Morten Svendsen, Trygve Solberg, Jørgen Ødegård

**Affiliations:** Institute of Animal and Aquacultural Sciences, Norwegian University of Life Sciences, Ås, Norway; GENO SA, Holsegata 22, 2317 Hamar, Norway; Aqua Aqua Gen AS, P.O. Box 1240, Sluppen, 7462 Trondheim, Norway

## Abstract

**Background:**

In dairy cattle, current genomic predictions are largely based on sire models that analyze daughter yield deviations of bulls, which are derived from pedigree-based animal model evaluations (in a two-step approach). Extension to animal model genomic predictions (AMGP) is not straightforward, because most of the animals that are involved in the genetic evaluation are not genotyped. In single-step genomic best linear unbiased prediction (SSGBLUP), the pedigree-based relationship matrix **A** and the genomic relationship matrix **G** are combined in a matrix **H**, which allows for AMGP. However, as the number of genotyped animals increases, imputation of the genotypes for all animals in the pedigree may be considered. Our aim was to impute genotypes for all animals in the pedigree, construct alternative relationship matrices based on the imputation results, and evaluate the accuracy of the resulting AMGP by cross-validation in the national Norwegian Red dairy cattle population.

**Results:**

A large-scale national dataset was effectively handled by splitting it into two sets: (1) genotyped animals and their ancestors (i.e. GA set with 20,918 animals) and (2) the descendants of the genotyped animals (i.e. D set with 4,022,179 animals). This allowed restricting genomic computations to a relatively small set of animals (GA set), whereas the majority of the animals (D set) were added to the animal model equations using Henderson’s rules, in order to make optimal use of the D set information. Genotypes were imputed by segregation analysis of a large pedigree with relatively few genotyped animals (3285 out of 20,918). Among the AMGP models, the linkage and linkage disequilibrium based **G** matrix (**G**_**LDLA0**_) yielded the highest accuracy, which on average was 0.06 higher than with SSGBLUP and 0.07 higher than with two-step sire genomic evaluations.

**Conclusions:**

AMGP methods based on genotype imputation on a national scale were developed, and the most accurate method, G_LDLA0_BLUP, combined linkage and linkage disequilibrium information. The advantage of AMGP over a sire model based on two-step genomic predictions is expected to increase as the number of genotyped cows increases and for species, with smaller sire families and more dam relationships.

**Electronic supplementary material:**

The online version of this article (doi:10.1186/s12711-015-0159-8) contains supplementary material, which is available to authorized users.

## Background

Genomic selection in dairy cattle is currently largely based on sire models, in which daughter yield deviations (DYD) or deregressed estimated breeding values (EBV) are used as data for the genomic evaluation [1]. This results in a two-(or more)step evaluation, where first the DYD or deregressed EBV are estimated using a traditional pedigree-based evaluation, and second, genomic estimates of breeding values (GEBV) are determined, which may be followed by a third step where the traditional EBV and GEBV are weighed and combined, e.g. [2]. Moving towards animal model genomic predictions (AMGP) seems the natural way forward, for which all data could be combined in a single evaluation. This would also promote the use of genomic predictions in other species for which sire models are less suited, because their family structures are less dominated by large sire families. However in this case, all animals involved in the prediction need to be genotyped. With the advent of increasingly more cost-effective genotyping methods, this may become a possibility for the future, but for now AMGP has to rely on pedigree, in addition to marker information.

In single-step genomic best linear unbiased prediction (SSGBLUP), information on (few) genotyped animals and (many) non-genotyped, but pedigree-recorded, animals is combined to yield one overall relationship matrix (**H**) [1, 3, 4], which can subsequently be used for BLUP of breeding values. In brief, SSGBLUP consists of: (1) starting from the pedigree relationship matrix (**A**), replace the relationship matrix of the genotyped animals by their genomic relationship matrix (**G**); and (2) predict the effects of the changes in relationship due to the introduction of **G** in step (1) for the relationships of the ungenotyped animals. A central assumption of SSGBLUP is that marker genotypes influence ungenotyped individuals via the pedigree-based relationship matrix **A**. Implicitly, SSGBLUP imputes the genotypes of the ungenotyped animals by using the **A** matrix-based regression coefficients **A**_**12**_**A**_**22**_^**−1**^, where 1 denotes the ungenotyped and 2 the genotyped set of animals [4]. Some illogical results due to the use of **A** matrix-based regressions have been reported [5]. More accurate genotype imputation methods exist, e.g. [6–9], and it is expected that such methods will become increasingly more appropriate as more genotypic data accumulate. Thus, our aim was to impute genotypes for all animals in the pedigree, construct alternative relationship matrices based on the imputation results, and evaluate the accuracy of the resulting AMGP by cross-validation in the national Norwegian Red dairy cattle population.

## Methods

### Phenotypic and pedigree data

Phenotypes on kg milk, kg fat, kg protein and somatic-cell-count (SSC) were kindly provided by GENO SA (http://www.geno.no) from their 2013 national routine evaluations consisting of 6,734,794 lactations on 3,274,518 Norwegian Red cows. The cows and bulls were linked by a pedigree containing 4,043,097 entries. The pedigree depth was truncated to five generations back from the genotyped bulls in order to limit computation costs. This national dataset was analyzed by the following single-trait repeatability animal model:

1 $${\mathbf{y}} = {\boldsymbol{\upmu}} + {\mathbf{Mm}} + {\mathbf{Fa}} + {\mathbf{Kd}} + {\mathbf{Xh}} + {\mathbf{Zp}} + {\mathbf{Zu}} + {\mathbf{e}},$$

where **y** is a vector of phenotypes (kg milk, kg fat, kg protein, or SSC); **m** is a vector of fixed month × year effects with the design matrix **M**; **a** is a vector of fixed age × lactation number effects with the design matrix **F**; **d** is a vector of fixed effects of days open with the design matrix **K**; **h** is a vector of random herd × year effects with the design matrix **X**; **p** is a vector of random permanent environmental effects with the design matrix **Z**; **u** is a vector of random animal effects with the same design matrix **Z**; and **e** is a vector of random errors. All random effects are assumed independently distributed, except **u** which has variance Var(**u**) = **G**_**x**_σ_u_^2^, where **G**_**x**_ denotes the relationship matrix between the animals that is varied as described below. Model (1) is the same as that used for GENO’s routine evaluations, except that genetic group effects were not fitted in the current evaluations. Variance components and trait heritabilities were the same as those assumed in the national evaluation (Table 1).

**Table 1:** Trait heritabilities (h^2^) and variance components of the random effects in the national evaluation

	Animal	Permanent environmental effect	Herd × year	Error	h^2^
kg_milk (×10^6^)	0.25	0.245	0.346	0.454	0.263
kg_fat	367	470	808	1052	0.194
kg_prot	183	264	459	444	0.205
SSC	0.137	0.319	0.052	0.554	0.136

### Genotypic data

Genotypes were provided by GENO SA on a total of 3438 Norwegian Red bulls, of which 1722 were genotyped using the 54 K Illumina BeadChip [10], and 2572 bulls were genotyped using the 25 K Affymetrix chip [11]. The genotypes of these 2572 bulls were imputed up to 54 K and were subsequently treated as true genotypes (856 bulls were genotyped by both chips). Genotyping, industry quality controls [individual call rate ≥97 %; Mendelian error rate of single nucleotide polymorphisms (SNPs) <2.5 %, SNP genotype call rate >25 %, and minor allele frequency (MAF) >0.05] and genotype imputation were performed by CIGENE (http://www.cigene.no), and resulted in 48,249 informative SNPs on 29 autosomes. The genotyped bulls were also used by GENO SA for their reference population in routine genomic predictions.

### Subsets of the data

Due to the size of the data, the total data was split into two sets: (1) the GA set contained all ancestors of the genotyped animals (truncated to five generations back) including the genotyped animals themselves, i.e. 20,918 animals, and (2) the D set contained all other animals, i.e. mostly descendants of the genotyped animals, i.e. 4,022,179 animals. This subdivision of the data made it possible to set up a (genomic) relationship matrix for the GA set and its inverse, which was calculated in parallel, at reasonable computational costs by LAPACK routines (because of the limited size of the GA set). Next, this inverse relationship matrix was augmented with the animals in the D set using Henderson’s rules for setting up the inverse of the pedigree-based relationship matrix [12], which is justified in Additional file 1. The inverse of the pedigree-based relationship matrix was also set up in this way (after confirmation that it yielded the same EBV as a standard BLUP evaluation).

Another subdivision of the data was used to test the accuracy of genomic selection. To this end, all lactations of animals born before January 1st 2007 were included in a training set (TRAIN set that included 6,732,765 lactations on 2,954,395 cows). The bulls born after January 1st 2007 and before December 31st 2008 were included in a validation set if they had more than 100 daughters with lactations (VAL set that included 153 bulls). DYD of these and all other bulls were estimated by DMU [13] using the complete or the TRAIN dataset and pedigree relationships. Distributions of the genotyped bulls over the TRAIN and VAL sets and over their birth-years are in Table 2. For evaluations based on the sire model, DYD of 2815 genotyped bulls were used for training (the remaining genotyped bulls did not have a sufficient number of daughters in the TRAIN dataset).

**Table 2:** Distribution of the genotyped bulls with sufficiently accurate DYD across the training (TRAIN) and validation (VAL) datasets and across their years of birth

Set	Birth (year)	Number
TRAIN	1964–1975	8
TRAIN	1976–1985	566
TRAIN	1986–1995	1244
TRAIN	1996–2000	592
TRAIN	2001	106
TRAIN	2002	102
TRAIN	2003	100
TRAIN	2004	93
TRAIN	2005	4
VAL	2007	101
VAL	2008	52
Total		2968

### Relationship matrices

ABLUP breeding value estimates (EBV) were obtained by fitting the pedigree-based relationship matrix, **A**., i.e. assuming Var(**u**) = **A**σ_u_^2^. G_LA1_BLUP EBV were obtained by fitting a linkage analysis based relationship matrix, **G**_**LA1**_, [14, 15] for which the probabilities of paternal/maternal inheritance were obtained using the LDMIP program [6]. Thus, G_LA1_BLUP denotes that the inverse relationship matrix **G**_**LA1**_^**−1**^ was calculated for the 20,918 animals in the GA set, and **G**_**LA1**_^**−1**^ was augmented with the animals in the D set using Henderson’s rules. The same strategy was used for the other relationship matrices described below. These large relationship matrices were fitted by the Mix99 package [16] using model (1) and the variance components as indicated in Table 1.

Preliminary analyses with the LDMIP program revealed that it converged to very extreme probabilities of paternal or maternal inheritance for some ungenotyped parts of the data, i.e. the information from the closely linked loci resulted in overconfident inheritance patterns for ungenotyped animals. To alleviate this problem, we also used an option in LDMIP that allows to assume that the loci are unlinked, in which case LDMIP reduces to the original iterative peeling algorithm [17, 18]. The paternal/maternal inheritance probabilities were assumed to equal 50/50 a priori (as in iterative peeling), which resulted in the **G**_**LA0**_ relationship matrix and G_LA0_BLUP-EBV.

In addition to probabilities of paternal or maternal inheritance, the LDMIP program yields genotype probabilities based on linkage analysis for all the animals in the GA set, which are equivalent to the actual genotypes of the genotyped animals. We used these genotype probabilities to set up a genomic relationship matrix at the gametic level, i.e. for both the paternal and maternal gamete of each animal in the GA set (two entries per animal):

2 $${\mathbf{G}} = {\mathbf{WW^{\prime}}}/\sum\nolimits_{j} {{\text{p}}_{\text{j}} ( 1- {\text{p}}_{\text{j}} ),}$$

where **G** is a (2n × 2n) matrix of gametic relationships (n = number of animals); **W** is a (2n × m) matrix of standardized genotypes (m = number of markers), i.e. element W_ij_ is the probability of a ‘1’ allele of gamete i at marker j expressed as a deviation from its mean, which is the frequency of the ‘1’ allele, p_j_. If E(W_ij_) = 0, the expectation of W_ij_^2^ equals Var(W_ij_) = p_j_(1 − p_j_). Because each allele in gamete i is a sample/copy of an allele in the founder population, the p_j_ should be equal the founder population allele frequencies such that E(W_ij_) = 0. If E(W_ij_) = 0, E(W_ij_^2^) = p_j_(1 − p_j_) holds even if the animal (or population) that encompasses gamete i is (completely) inbred. In this study, we did not attempt to estimate founder population frequencies, and p_j_ was calculated as the allele frequencies of the loci in the TRAIN population.

The relationship of a gamete with itself is 1. Thus, the diagonals of **G** are expected to equal 1, because E(W_ij_^2^) = p_j_(1 − p_j_), but will deviate from 1 due to (a) sampling, and (b) the use of genotype probabilities instead of actual genotypes, which are less variable [smaller E(W_ij_^2^)] than actual genotypes. The latter results in the elements G_ii_ = Σ_j_W_ij_^2^/Σ_j_p_j_(1 − p_j_) being substantially underestimated, due to the uncertainty of the genotypes. If gametes i and j both had diagonal elements that were too small, G_ii_ < 1 and G_jj_ < 1, then their relationship G_ij_ is also expected to be underestimated, which is corrected here by adding ${{\tilde{A}}}_{ij} \sqrt {(1 - G_{ii} )(1 - G_{jj} )}$ to G_ij_, where ${{{\tilde{\varvec{A}}}}}$ denotes the pedigree-based gametic relationship matrix.

Due to the above point (a), G_ii_ and G_jj_ may be greater than 1, and we assumed that G_ij_ was overestimated due to sampling. In this case, we scaled the relationship estimate back to $G_{ij} /\sqrt {G_{ii} G_{jj} }$ in order to correct for this sampling error. Another possibility is that G_ii_ is greater than 1 and G_jj_ less than 1, in which case, we were uncertain about the over- or underestimation of G_ij_ and left it unchanged.

The corrections of the **G** matrix mentioned above may be summarized in matrix form by:

3 $${\mathbf{G}}_{{{\mathbf{LDLA}}1}} = {\mathbf{S}}({\mathbf{DGD}} + \Delta {{\tilde{\varvec{A}}}} \Delta ){\mathbf{S^{\prime}}}/2,$$

where **D** is a diagonal matrix with elements $\sqrt {1/({\text{G}}_{\text{ii}} )}$ when G_ii_ is greater than 1, or 1 elsewhere, **Δ** is a diagonal matrix with elements $\sqrt {(1 - {\text{G}}_{\text{ii}} )}$ when G_ii_ less than 1, or 0 elsewhere, and **S** is a design matrix that indicates which gametes belong to which animals, which reduces the gametic relationship matrix ${\mathbf{DGD}} + \Delta{{\tilde{\varvec{A}}}}\Delta$ to an animal relationship matrix of size number of animals squared. Additional file 2 presents a small example on the calculation of Eqs. (2) and (3). In cases where old ancestors are not genotyped, Eq. (2) uses linkage analysis to estimate their genotype probabilities, and if genotype probabilities become too uncertain, Eq. (3) adds pedigree relationships to the relationships based on genotype probabilities. It should be noted that the above matrix manipulations leave the resulting matrix (semi)positive definite if the **G** and ${{\tilde{\varvec{A}}}}$ matrices are (semi)positive definite. This relationship matrix is called ‘LDLA’ because it combines linkage (from linkage analysis) and linkage disequilibrium (from identity of marker alleles) information. The above relationship matrix can also be setup without using information from neighboring loci in the LDMIP analysis, in which case it will be called **G**_**LDLA0**_, resulting in G_LDLA0_BLUP-EBV.

A commonly used AMGP method is SSGBLUP, which uses the **H** matrix [1, 3]. We used SSGBLUP as implemented in DMU [13], using the G-ADJUST option which adjusts elements in the genomic relationship so that the average of diagonal elements and the average of the off-diagonal elements equal their corresponding averages in the **A** matrix for the genotyped animals. SSGBLUP requires the genomic relationship matrix of the genotyped animals which was calculated as in [19], i.e.:

$${\mathbf{G}}_{{\mathbf{T}}} = {\mathbf{W}}_{{\mathbf{T}}} {\mathbf{W}}_{{\mathbf{T}}}^{\prime } /\sum\nolimits_{j} { 2 {\text{p}}_{\text{j}} ( 1- {\text{p}}_{\text{j}} ),}$$

where **W**_**T**_ is a matrix of standardized genotypes, with elements W_Tij_ denoting the number of ‘1’ alleles of animal i at marker j expressed as a deviation from its mean, 2p_j_.

We compared the above methods based on an animal model to methods based on a sire model (SM), for which only the genotyped bulls in the TRAIN dataset (Table 2) and their DYD were used as (unweighted) data records. SM-GBLUP uses the genomic relationship matrix **G**_**T**_, and variance components were estimated within the data (since the variance components in Table 1 do not apply to DYD). We also applied SM-ABLUP, which is the same as SM-GBLUP except that the genomic relationship matrix is replaced by the pedigree-based relationship matrix **A**.

## Results

Table 3 shows the correlations between 2013 DYD and 2007 EBV within the VAL set of bulls for the methods based on a two-step sire model (standard errors are from 10,000 bootstrapping samples [20]). In addition, Table 3 includes the accuracies of EBV estimated as the correlation between EBV and DYD relative to the maximum correlation between perfect EBV predictions and DYD, which equals the square root of the reliability of the DYD. The latter was calculated as the average of R^2^ = d_e_/(d_e_ + α) for the VAL bulls, where d_e_ is the effective number of daughters of each bull (as provided by DMU) and α = (4 − h^2^)/h^2^. For all traits, GEBV were more accurate than EBV based on the **A** matrix and differences were statistically significant as tested by the Hotelling–Williams test for dependent correlations [21]. In spite of the high standard errors on the correlation estimates, the Hotelling-Williams test yielded significant results, due to the dependencies between the correlations (the DYD used were the same as those for the tested correlations). On average, there is a difference in accuracy of 0.10 between SM-GBLUP and SM-ABLUP (0.08, 0.10, 0.13, and 0.10 for milk, fat and protein yield and SSC, respectively).

**Table 3:** Correlations between 2013 DYD and 2007 EBV (±SE) and accuracy of EBV predicted for a set of young evaluation bulls when using the bulls of the TRAIN set for training

Method^a^	kg_milk	kg_fat	kg_prot	SSC
Correlations between EBV and DYD^b^				
SM-ABLUP	0.404 ± 0.071*	0.466 ± 0.064*	0.396 ± 0.070**	0.355 ± 0.078**
SM-GBLUP	0.48 ± 0.068	0.561 + 0.057	0.514 ± 0.061	0.445 ± 0.063
Accuracies of EBV^c^				
SM-ABLUP	0.421	0.493	0.417	0.384
SM-GBLUP	0.500	0.593	0.542	0.481

^a^SM-ABLUP and SM-GBLUP use the **A** and **G**_**T**_ matrix, respectively

^b^A significant reduction of SM-ABLUP relative to SM-GBLUP is indicated by * (P < 0.05), ** (P < 0.01)

^c^Accuracy = Corr((G)EBV,DYD)/$\sqrt {({\text{R}}^{ 2} \,{\text{of}}\;{\text{DYD}})}$

**Fig. 1:**
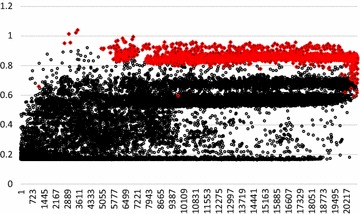
Diagonal elements of the paternal alleles of the **G** matrix (Eq. (2)). Elements of genotyped animals are marked in *red*. Elements are sorted from old to young animals

Figure 1 shows the diagonal elements of the paternal alleles of the **G** matrix (the maternal alleles show a very similar pattern; result not shown here). The values of the diagonals from the genotyped bulls are on average 0.86, i.e. substantially less than 1. This is probably because the iterative peeling algorithm has to estimate probabilities for the paternal allele being “1” or “0” for heterozygous loci, which results in the variance of alleles to be on average less than p_j_(1 − p_j_). For the old ancestors, many diagonal elements are very low, whereas for the more recent ungenotyped animals most of the diagonals elements are between 0.6 and 0.7. Thus, the corrections to the **G** matrix applied in Eq. (3) resulted in substantial additions from the **A** matrix, especially for old ancestors, and very few downward corrections of the elements of **G** (few diagonals were >1).

Table 4 shows the correlation between 2013 DYD and 2007 EBV and their accuracy when national animal models are used for evaluation, using population-wide relationship matrices (**A**, **G**_**LA**_, **G**_**LDLA**_ or **H**). When moving from SM-ABLUP to ABLUP, the accuracy increases on average by only 0.02 (Tables 2, 3). When moving from SM-GBLUP to SSGBLUP, the accuracy increases on average by 0.01. This small increase in accuracy is in line with results on SSGBLUP in the literature [1]. The family structure of dairy cattle, which is dominated by large sire families, makes sire model evaluations quite accurate.

**Table 4:** Correlations between 2013 DYD and 2007 EBV (±SE) and accuracies of EBV predicted for a set of young evaluation bulls using all records on cows born before January 1st 2007 for training

Method	kg_milk	kg_fat	kg_prot	SSC
Correlations between EBV and DYD^a^				
ABLUP	0.413 ± 0.066**	0.460 ± 0.065**	0.423 ± 0.067**	0.390 ± 0.071**
SSGBLUP	0.497 ± 0.073*	0.585 ± 0.058*	0.518 ± 0.063^-^	0.434 ± 0.070**
G_LA1_BLUP	0.319 ± 0.067**	0.272 ± 0.078**	0.370 ± 0.064**	0.284 ± 0.078**
G_LA0_BLUP	0.432 ± 0.065**	0.465 ± .065**	0.440 ± 0.066**	0.377 ± 0.072**
G_LDLA1_BLUP	0.522 ± 0.061^-^	0.525 ± 0.064**	0.476 ± 0.065*	0.529 ± 0.053^-^
G_LDLA0_BLUP	0.555 ± 0.057	0.633 ± 0.047	0.543 ± 0.059	0.538 ± 0.054
Accuracies of EBV^b^				
ABLUP	0.430	0.486	0.445	0.422
SSGBLUP	0.518	0.618	0.546	0.469
G_LA1_BLUP	0.332	0.288	0.390	0.307
G_LA0_BLUP	0.450	0.492	0.464	0.408
G_LDLA1_BLUP	0.543	0.555	0.501	0.572
G_LDLA0_BLUP	0.578	0.669	0.572	0.581

^a^A significant reduction relative to G_LDLA0_BLUP is indicated by * (P < 0.05), ** (P < 0.01), and ^-^ (not significant)

^b^Accuracy = Corr(EBV,DYD)/$\sqrt {({\text{R}}^{ 2} \,{\text{of}}\;{\text{DYD}})}$

The methods based on linkage analysis, G_LA1_BLUP and G_LA0_BLUP, resulted in lower accuracies than SM-GBLUP and SSGBLUP, for all traits. G_LDLA1_BLUP was more accurate than SM-GBLUP and SSGBLUP for two of the four traits. G_LDLA0_BLUP was more accurate than G_LDLA1_BLUP, SM-GBLUP and SSGBLUP for the four traits. G_LDLA0_BLUP was on average 0.06 more accurate than SSGBLUP. The increased accuracy obtained with G_LDLA0_BLUP compared to the other methods was statistically significant, except for kg_milk and SSC, for which G_LDLA0_BLUP was not significantly more accurate than G_LDLA1_BLUP.

Table 5 shows the regression coefficients of the 2013 DYD on the 2007 EBV in the VAL set (standard errors are based on 10,000 bootstrap samples) to estimate biases of the different methods. In the absence of selection, this regression coefficient is expected to be 1 for unbiased EBV. Overall, standard errors were large and regression coefficients tended to be less than 1 (even for methods that are theoretically known to be unbiased such as ABLUP and G_LA0_BLUP). Apart from the methods with poor multi-locus linkage analysis (G_LA1_BLUP and G_LDLA1_BLUP), and SSGBLUP for the SSC-trait, the regression coefficients did not significantly deviate from 1.

**Table 5:** Regression coefficients of DYD on EBV (±SE) predicted for a set of young evaluation bulls

Method	kg_milk	kg_fat	kg_prot	SSC
ABLUP	0.975 ± 0.174	0.906 ± 0.138	0.944 ± 0.170	0.787 ± 0.141
SSGBLUP	0.812 ± 0.142	0.892 ± 0.112	0.805 ± 0.119	0.643 ± 0.113
G_LA1_BLUP	0.489 ± 0.103	0.395 ± 0.113	0.538 ± 0.099	0.445 ± 0.122
G_LA0_BLUP	0.994 ± 0.165	0.907 ± 0.136	0.962 ± 0.164	0.760 ± 0.143
G_LDLA1_BLUP	0.765 ± 0.107	0.758 ± 0.105	0.647 ± 0.108	0.816 ± 0.103
G_LDLA0_BLUP	0.913 ± 0.110	0.980 ± 0.089	0.848 ± 0.109	0.827 ± 0.104
SM-ABLUP	1.237 ± 0.239	1.079 ± 0.127	1.098 ± 0.226	0.858 ± 0.182
SM-GBLUP	1.032 ± 0.165	1.094 ± 0.161	1.121 ± 0.156	0.802 ± 0.127

## Discussion

Novel genomic prediction methods using an imputation-based animal model, such as G_LDLA0_BLUP, were developed and tested, for which imputation of genotype probabilities was used for ungenotyped animals in order to account for inaccuracies that would occur if actual genotypes were imputed. Because of the uncertainty of genotype probabilities, their use resulted in underestimated relationships and this was most apparent for the self-relationships (diagonal elements of the relationship matrix). This was corrected by adding proportions of the **A** matrix such that the diagonal elements of the gametic relationship matrix were equal to their expectation of 1, and the off-diagonal elements were also increased by these proportions (since when the variance of the genotypes is underestimated by genotype probabilities, their covariance is also expected to be underestimated). This resulted in a genomic relationship matrix that combined linkage and linkage disequilibrium information, and yielded higher genomic prediction accuracies than the alternative methods studied here.

LDMIP was used for genotype imputation. Alternative imputation software methods (e.g. [7–9]) could be used as long as they: (1) impute genotypes for ungenotyped animals (this requires the use of pedigree data), and (2) yield genotype probabilities instead of actual genotypes in order to reflect the uncertainty in the genotype estimates. Although it has been reported that **G** matrices based on linkage analysis using LDMIP resulted in high accuracies [15, 22], in the large-scale application that we developed here with few genotyped animals relative to the total number of animals, the multi-locus iterative peeling algorithm in LDMIP seemed to severely overestimate the information content contained in the closely linked marker data. The assumption of unlinked loci implies that the inheritance patterns of the loci become less dependent on each other, thereby resulting in effectively more independent loci for the ungenotyped animals, and, when averaged over many loci, more accurate estimates of relationships. It may be expected that, in the future, many animals will be genotyped, and thus inheritance patterns become more certain. The imputation-based prediction methods perform better in situations with many genotyped relative to ungenotyped animals as shown in [15, 22]. In our data, this was not the case, but, for all traits, G_LDLA0_BLUP was still more accurate than any of the alternative methods considered.

Using the animal model, for genomic prediction of national EBV, it was necessary to split the data into two sets: (1) the genotyped and their ancestors (GA set) and their ungenotyped descendants (D set). In our data, the brute-force inversion of the genomic relationship matrices for the GA set was possible by parallel computation. When it will become possible to genotype many cows, the GA set may consist of more than 100,000 animals, and thus, this inversion may become problematic, in which case, methods that can invert large **G** matrices [23] or avoid the inversion of **G** are needed. Inversion of **G** can be avoided [3, 24, 25], for instance by solving the EBV of the animals in the GA set by Henderson’s alternative mixed model equations for singular **G** [12], and solving the EBV of the animals in the D set by the iteration on the data approach [26]. The large number of animals in the D set was augmented to this **G** matrix using Henderson’s rules for the inversion of **A**. The genetic evaluation models for dairy traits, which were used here, were rather simple, and more complicated (multi-trait random regression) models could be applied in practice. However, since the presented alternative models are all based on changes of the relationship matrix between the animals, and these more complicated genetic evaluation models are also based on relationship matrices or their inverses, it is rather straightforward to apply the current developments to these more complicated evaluation models.

For all the traits considered here, G_LDLA0_BLUP yielded higher prediction accuracies than SSGBLUP. This may be due mainly to the assumption in SSGBLUP that ungenotyped animals have 50/50 inheritance patterns, which leads, for example, to an increased genomic relationship between sibs that is explained by increased relationships between their parents. In segregation analysis, such as performed by LDMIP, the similarity between sibs is explained by the co-inheritance of the same alleles from their parents. This projection of current genomic relationships towards the relationships between founder animals by SSGBLUP will be especially unrealistic for deep pedigrees, in which many alternative inheritance patterns may explain low or high genomic relationships between animal pairs, and founder animals are separated by many generations from the current animals. In addition, the scaling of the **A** and **G** matrices may affect accuracies of SSGBLUP [1, 22]. Here, a standard method provided in DMU was used [13].

Equation (3) combines **A** and **G** matrix elements into an overall **G**_**LDLA1**_ matrix. This combination of **A** and **G** matrix elements is known to be problematic based on the SSGBLUP theory, because of differences in the definition of the founder populations that underlie the two relationship matrices. Ideally, the allele frequencies used to calculate the **G** matrix should be estimated in the founder population used for the **A** matrix, but this was not attempted here, although the segregation analysis can estimate founder population allele frequencies [17]. The founder population (as used for the **A** matrix) is not a well-defined population since it consists of all animals with unknown parents that range from the oldest ancestors to quite recent animals. For the calculation of the **G** matrix, allele frequencies as estimated in the genotyped bull population were used, which defines them as the founder population for this matrix [27]. This population of bulls stretches over many years, which also makes it a poorly defined founder population. In future research, we intend to improve the G_LDLA0_BLUP method by using segregation analysis to estimate allele frequencies in the founder population, and in the absence of a single founder population, to split the founder population into several genetic groups and estimate the allele frequencies within each of these, in order to extend the G_LDLA0_BLUP approach to an animal model with genetic groups effects [28].

The dairy pedigree considered here was not very deep (five generations). In other situations or species, pedigrees may be deeper which has several consequences: (1) it increases the computational costs substantially since the size of the GA set increases as the number of ancestors in the pedigree increases; LDMIP computations increase approximately linearly with the number of animals in the GA set (instead of with the number of genotyped animals), and computation efforts to obtain the **G** inverse increase with the power 3 of the number of GA animals; and (2) the genotype probabilities of old ancestors of genotyped animals will become close to Hardy–Weinberg frequencies, i.e. there is hardly any information to differentiate their genotypes; this results in scaled genotypes, W_ij_, close to 0, and thus G_ii_ = Σ_j_W_ij_^2^/Σ_j_p_j_(1 − p_j_) values close to 0 and **G**_**LDLA0**_ matrix elements of such old ancestors will be close to **A** matrix elements, i.e. the $\Delta{\tilde{\varvec{A}}} \Delta$ term of Eq. (3) becomes larger where **Δ** reflects the inaccuracy of the estimation of W_ij_. Since it is known from pedigree-based breeding that phenotypes on old (ungenotyped) ancestors hardly contribute to the accuracy of the EBV of the current animals, the same may be expected from **G**_**LDLA0**_-based EBV. However, in the **G**_**LDLA0**_ case, the ancestors have to be older before this happens, because as long as the iterative peeling can predict genotypes with any accuracy, the **G**_**LDLA0**_ matrix can make better use of the information on ungenotyped ancestors than the **A** matrix. Thus, old ungenotyped ancestors are expected to contribute little to the accuracy of current genotyped animals, but more than in the case of ABLUP.

The animal model ABLUP yielded on average only 0.02 more accurate results than the sire model SM-ABLUP (Tables 3, 4). This relatively good accuracy of the sire model is probably specific to the dairy cattle situation where large sire families dominate the population structure. In other species, for which dam families are more important (e.g. pigs and poultry), the sire model will not fit so well, and the advantage of the genomic selection methods based on an animal model will increase compared to the dairy cattle situation. The introduction of genotyped cows in dairy cattle breeding will make GS based on a sire model less suitable, because genotyped cows can only be included as ‘bulls with very inaccurate DYD’ (i.e. phenotypes) in SM-GBLUP. In GS based on a sire model, the weighing of these alternative information sources (real DYD and phenotypes) will be delicate, with a risk of double counting records (methods should be used that avoid double counting). In AMGP models, all bull and cow data are ‘automatically’ combined and thus, it is expected that they will become more suitable in the future.

All the models used here were based on relationship matrices, and therefore implicitly assumed normally distributed allelic effects. The **G**_**LDLA0**_ matrix (Eq. (3)) consists of two parts: one part that is due to marker genotypes (probabilities) and one part that is due to pedigree relationships. The part that is due to marker genotypes (probabilities) could be analyzed by a nonlinear SNP-based model, such as BayesB [28], while simultaneously fitting a polygenic effect into the model with relationship matrix ${\mathbf{S}}\Delta \mathop {\mathbf{A}}\limits^{\sim } \Delta {\mathbf{S^{\prime}}}/2$ (from Eq. (3)). In this way, a BayesB-type of analysis could be implemented in a (national) animal model setting.

## Conclusions

Animal model genomic prediction methods based on genotype imputation on a national scale were developed, and the most accurate method, G_LDLA0_BLUP, combined linkage and linkage analysis information. G_LDLA0_BLUP yielded on average a 0.06 higher accuracy than SSGBLUP and a 0.07 higher accuracy than GBLUP based on a sire model. The latter advantage is expected to increase as the number of genotyped cows increases and also for species, with smaller sire families and stronger dam relationships, for which the use of animal models is crucial.

## Additional files

10.1186/s12711-015-0159-8 Henderson’s rules for the inverse of the numerator relationship matrix when parental relationships are (partly) based on SNPs. Proof that Henderson’s rules hold when parental relationships are based on SNPs.
10.1186/s12711-015-0159-8 Example of the calculation of the G_LDLA_ matrix. This file describes an example on how to calculate the G_LDLA_ matrix.
