# Supplementary material for: Genomic predictions based on animal models using genotype imputation on a national scale in Norwegian Red cattle
Source: Genet Sel Evol. 2015 Oct 13;47:79. doi: 10.1186/s12711-015-0159-8 (PMC4605129; doi:10.1186/s12711-015-0159-8)
Supplement: Supplementary file 1 — 10.1186/s12711-015-0159-8 Henderson’s rules for the inverse of the numerator relationship matrix when parental relationships are (partly) based on SNPs. Proof that Henderson’s rules hold when parental relationships are based on SNPs. [file 12711_2015_159_MOESM1_ESM.docx]

**Henderson’s rules for the inverse of the numerator relationship matrix when parental relationships are (partly) based on SNPs**

**Henderson’s rules to set up A^-1^ when A is a pedigree based relationship matrix**: Many alternative derivations of Henderson’s rules exist (e.g. [1-3]). We will briefly describe one version here that is most readily extended to situations where parental relationships may not be based on pedigree. Application of Henderson’s rules requires that the animals are sorted from old to young, such that parents precede their offspring when evaluating animals 1, 2, .. N. It may be noted that, when evaluating animal *i*, the inverse of the relationship matrix **A** of animals 1..(*i*-1) exists, i.e. (**A_11_**)^-1^ is available where subscript (11) denotes the block of **A** of animals 1..(*i*-1). If only animals 1..(*i*-1) would have been available, the algorithm would actually have stopped after evaluating animal (*i*-1), and would have produced (**A_11_**)^-1^. Key to the algorithm is to write the genetic value of animal *i*, *u_i_*, as

*u_i_* = ½*u_s_* + ½*u_d_* + δ_i_, (S1)

where *s* and *d* represent the parents of *i*, and δ_i_ is the Mendelian sampling deviation of animal *i*. If we assume a genetic variance of 1, and in the absence of inbreeding, V(δ_i_)=½ (for situations with inbreeding see [2]). Following [3] we may write the set of equations:

**u_1_** = **δ_1_**

u_i_=**t’u_1_** + δ_i_

where the first equation defines the genetic values of the block 1 animals, **u_1_**, to equal **δ_1_**, with V(**δ_1_**)=**A_11_**; and **t’u_1_**,= ½*u_s_* + ½*u_d_*, i.e. **t** is a vector with ½‘s at the positions of the parents of *i*, and zeros elsewhere. In matrix form these equations are:

$$\left[ \begin{matrix} \boldsymbol{u}_{1} \\ u_{i} \end{matrix} \right]=\left[ \begin{matrix} 0 & 0 \\ \boldsymbol{t'} & 0 \end{matrix} \right]\left[ \begin{matrix} \boldsymbol{u}_{1} \\ u_{i} \end{matrix} \right]+\left[ \begin{matrix} \boldsymbol{\delta}_{1} \\ \delta_{i} \end{matrix} \right]$$

Taking $\left[ \begin{matrix} \boldsymbol{u}_{1} \\ u_{i} \end{matrix} \right]$to the left-hand-side:

**Tu**=**δ**,

where $\boldsymbol{T=}\left[ \begin{matrix} \boldsymbol{I} & \boldsymbol{0} \\ \boldsymbol{-t'} & 1 \end{matrix} \right]$and $\boldsymbol{\delta}=\left[ \begin{matrix} \boldsymbol{\delta}_{1} \\ \delta_{i} \end{matrix} \right]$. Thus, **u**=**T^-1^δ**, and V(**u**) = **A** = **T^-1^**V(**δ**)**T’^-1^**, where

$V\left( \boldsymbol{\delta} \right)=\boldsymbol{D}=\left[ \begin{matrix} \boldsymbol{A}_{\boldsymbol{11}} & 0 \\ 0 & \frac{1}{2} \end{matrix} \right]$. (S2)

We need the inverse of **A**, i.e. **A^-1^**=**T’D^-1^T**, where:

$\boldsymbol{D}^{\boldsymbol{-1}}=\left[ \begin{matrix} \boldsymbol{A}_{\boldsymbol{11}}^{\boldsymbol{-1}} & 0 \\ 0 & 2 \end{matrix} \right]$,

And writing out **T’D^-1^T** gives:

$\boldsymbol{A}^{-1}=\left[ \begin{matrix} \boldsymbol{A}_{\boldsymbol{11}}^{\boldsymbol{-1}}+2\boldsymbol{tt'} & -2\boldsymbol{t} \\ -2\boldsymbol{t'} & 2 \end{matrix} \right]$ (S3)

From which Henderson’s rules to set up **A^-1^** can be found:

1. Add ½ to the positions (s,s), (s,d), (d,s), and (d,d) (due to the 2**tt**’ term in (S3));
2. Add -1 to the positions (s,i), (d,i), (i,s), and (i,d) (due to the -2**t** and -2**t**’ terms in (S3));
3. Add 2 to the position (i,i) (due to the diagonal 2 in (S3)).

The above are the rules when both parents of i are known. If only one parent is known (say s), equation (S1) reduces to: *u_i_* = ½*u_s_* + δ_i_, and δ_i_ includes the genetic effect of the unknown parent resulting in V(δ_i_)=¾., which results in (similar) rules for one unknown parent. When both parents are unknown, *u_i_* = δ_i_, and δ_i_ includes the total genetic effect: V(δ_i_)=1, which results in the row and column of *i* in (S3) having a 1 at the diagonal and 0’s elsewhere.

Starting with an identity matrix for the founder animals, the above rules can be applied to extend the **A^-1^** with animals *i*, *i*+1, .., N, until the **A^-1^** of all animals is found.

**Henderson’s rules to set up** $\boldsymbol{A}$**^*-1^ when some parental relationships are based on G:** Here, we assume that there is a set of ancestors, 1,..,(*i*-1) (called the GA-set in the main text), that have (partly imputed) genotypes, and their relationship matrix **G_11_** (including **G_11_^-1^**) is known from marker data. Next there is a set of descendants, *i*,..,N (offspring, grand-offspring, etc.) that are not genotyped, and to which Henderson’s rules will be applied here.

The key Equation (S1): *u_i_* = ½*u_s_* + ½*u_d_* + δ_i_, partitions the total genetic effect into a term due to the sire, a term due to the dam and a Mendelian sampling term. This partitioning is not affected by whether the sire, the dam or both are genotyped or not. However, if animal *i* itself had been genotyped, the assumption that the Mendelian sampling term is independent of all other genetic effects, which is assumed in Equation (S2), would not hold. For instance, the markers might indicate that *i* has received more alleles from its granddam than from its grandsire. Thus, the assumption that the descendants *i*,..,N are not genotyped is essential to the application of Henderson’s rules.

Thus, if the relationship matrix of animals 1,..,(*i*-1) is **G_11_**, Equation (S2) becomes:

$\boldsymbol{D}=\left[ \begin{matrix} \boldsymbol{G}_{\boldsymbol{11}} & 0 \\ 0 & \frac{1}{2} \end{matrix} \right]$.

And the inverse of the augmented relationship matrix becomes:

$\boldsymbol{A}^{*-1}=\left[ \begin{matrix} \boldsymbol{G}_{\boldsymbol{11}}^{\boldsymbol{-1}}+2\boldsymbol{tt'} & -2\boldsymbol{t} \\ -2\boldsymbol{t'} & 2 \end{matrix} \right]$, (S4)

where superscript * indicates that relationships are partly due to markers and partly due to pedigree. It may be noted that the only difference between (S3) and (S4) is the substitution of **A_11_^-1^** by **G_11_^-1^**. The terms that resulted in the additions from Henderson’s rules (2**tt**’, -2**t**, -2**t**’, and 2) are identical in Equations (S3) and (S4). Thus, Henderson’s rules are identical, whether the inverse of the relationships of the ancestors of *i* is based on pedigree or on marker or on a combination of both information sources.

**References**

[1] Henderson C.R., A simple method for computing the inverse of a numerator relationship matrix used in prediction of breeding values. , Biometrics. **32** (1976) 69-79.

[2] Quaas R.L., Computing the diagonal elements of a large numerator relationship matrix., Biometrics. **32** (1976) 949-953.

[3] Quaas R.L., Additive genetic model with groups and relationships., J. Dairy Sci. **71** (1988) 1338-1345.
