# Supplementary material for: Genomic predictions based on animal models using genotype imputation on a national scale in Norwegian Red cattle
Source: Genet Sel Evol. 2015 Oct 13;47:79. doi: 10.1186/s12711-015-0159-8 (PMC4605129; doi:10.1186/s12711-015-0159-8)
Supplement: Supplementary file 2 — 10.1186/s12711-015-0159-8 Example of the calculation of the GLDLA matrix. This file describes an example on how to calculate the GLDLA matrix. [file 12711_2015_159_MOESM2_ESM.docx]

Supplementary material 2:

**Example of the calculation of the G_LDLA_ matrix**

The following yields a small example on how to set up a **G_LDLA_** matrix using Equations (2) and (3) in the main text. We assume a pedigree with 4 animals. Animals 1 and 2 are the founders of the pedigree and the sire and dam of animal 3, respectively. Animal 4 has animals 1 and 3 as sire and dam, respectively. There is only one marker with alleles “0” and “1”, the latter with allele frequency 0.25. Animals 3 and 4 are genotyped as heterozygote and homozygote “1 1“, respectively; the genotypes of animals 1 and 2 are missing. Segregation analysis yields the following probabilities for the “1” allele of the eight gametes:

[pat_1_ mat_1_ … pat_4_ mat_4_] = [0.6667 0.6667 0.2084 0.2084 0.8333 0.1667 1 1]

where pat_i_ (mat_i_) denotes the paternally (maternally) derived gamete of animal i. The standardized genotypes **W** (Equation (2)) are obtained by subtracting the allele-frequency (0.25) from the above probabilities:

**W**’=[0.4167 0.4167 -0.0416 -0.0416 0.5833 -0.0833 0.75 0.75]

And application of Equation (2) yields the gametic **G** matrix:

**G** =

| 0.9261 | 0.9261 | -0.0925 | -0.0925 | 1.2963 | -0.1851 | 1.6668 | 1.6668 |
| --- | --- | --- | --- | --- | --- | --- | --- |
| 0.9261 | 0.9261 | -0.0925 | -0.0925 | 1.2963 | -0.1851 | 1.6668 | 1.6668 |
| -0.0925 | -0.0925 | 0.0092 | 0.0092 | -0.1294 | 0.0185 | -0.1664 | -0.1664 |
| -0.0925 | -0.0925 | 0.0092 | 0.0092 | -0.1294 | 0.0185 | -0.1664 | -0.1664 |
| 1.2963 | 1.2963 | -0.1294 | -0.1294 | 1.8146 | -0.2591 | 2.3332 | 2.3332 |
| -0.1851 | -0.1851 | 0.0185 | 0.0185 | -0.2591 | 0.0370 | -0.3332 | -0.3332 |
| 1.6668 | 1.6668 | -0.1664 | -0.1664 | 2.3332 | -0.3332 | 3.0000 | 3.0000 |
| 1.6668 | 1.6668 | -0.1664 | -0.1664 | 2.3332 | -0.3332 | 3.0000 | 3.0000 |

The gametic pedigree relationship matrix is for this pedigree:

$\tilde{\boldsymbol{A}}$=

| 1 | 0 | 0 | 0 | 0.5 | 0 | 0.5 | 0.25 |
| --- | --- | --- | --- | --- | --- | --- | --- |
| 0 | 1 | 0 | 0 | 0.5 | 0 | 0.5 | 0.25 |
| 0 | 0 | 1 | 0 | 0 | 0.5 | 0 | 0.25 |
| 0 | 0 | 0 | 1 | 0 | 0.5 | 0 | 0.25 |
| 0.5 | 0.5 | 0 | 0 | 1 | 0 | 0.5 | 0.5 |
| 0 | 0 | 0.5 | 0.5 | 0 | 1 | 0 | 0.5 |
| 0.5 | 0.5 | 0 | 0 | 0.5 | 0 | 1 | 0.5 |
| 0.25 | 0.25 | 0.25 | 0.25 | 0.5 | 0.5 | 0.5 | 1 |

The elements of the diagonal **D** matrix can be derived from the diagonals of **G** as max(1/√G_ii_,1), i.e.:

**D** = diag(1, 1, 1, 1, 0.7424, 1, 0.5774, 0.5774).

The elements of the diagonal **Δ** matrix can be derived from the diagonals of **G** as √[max(1-G_ii_,0)], i.e.:

**Δ** = diag(0.2719, 0.2719, 0.9954, 0.9954, 0, 0.9813, 0, 0).

Using Equation (3) this results in the gametic relationship matrix:

$\boldsymbol{DGD}+\boldsymbol{\Delta}\tilde{\boldsymbol{A}}\boldsymbol{\Delta}$**=**

| 1 | 0.926074 | -0.09245 | -0.09245 | 0.962327 | -0.18513 | 0.962327 | 0.962327 |
| --- | --- | --- | --- | --- | --- | --- | --- |
| 0.926074 | 1 | -0.09245 | -0.09245 | 0.962327 | -0.18513 | 0.962327 | 0.962327 |
| -0.09245 | -0.09245 | 1 | 0.00923 | -0.09607 | 0.506873 | -0.09607 | -0.09607 |
| -0.09245 | -0.09245 | 0.00923 | 1 | -0.09607 | 0.506873 | -0.09607 | -0.09607 |
| 0.962327 | 0.962327 | -0.09607 | -0.09607 | 1 | -0.19237 | 1 | 1 |
| -0.18513 | -0.18513 | 0.506873 | 0.506873 | -0.19237 | 1 | -0.19237 | -0.19237 |
| 0.962327 | 0.962327 | -0.09607 | -0.09607 | 1 | -0.19237 | 1 | 1 |
| 0.962327 | 0.962327 | -0.09607 | -0.09607 | 1 | -0.19237 | 1 | 1 |

Pre- and post-multiplying with the design matrix **S**=[1 1 0 0 0 0 0 0;0 0 1 1 0 0 0 0; 0 0 0 0 1 1 0 0; 0 0 0 0 0 0 1 1] yields the final **G_LDLA_** matrix:

$\boldsymbol{G}_{\boldsymbol{LDLA}}=\boldsymbol{S}(\boldsymbol{DGD}+\boldsymbol{\Delta}\tilde{\boldsymbol{A}}\boldsymbol{\Delta}$**)S’**/2 =

The corresponding **H** matrix from the SSGBLUP method is in this example:

**H** =

**G_LDLA_** and **H** are clearly different in this single-marker example, and it seems that the elements of **G_LDLA_** are somewhat less variable.
